# Girls and Boys Have a Different Cardiometabolic Response to Obesity Treatment

**DOI:** 10.3389/fendo.2018.00579

**Published:** 2018-10-02

**Authors:** Marketta Dalla Valle, Tiina Laatikainen, Hanna Potinkara, Päivi Nykänen, Jarmo Jääskeläinen

**Affiliations:** ^1^Department of Pediatrics, North Karelia Central Hospital, Joensuu, Finland; ^2^Siun Sote - the Joint Municipal Authority for North Karelia Social and Health Services, Joensuu, Finland; ^3^Department of Pediatrics, University of Eastern Finland, Kuopio, Finland; ^4^Health Department, National Institute for Health and Welfare, Helsinki, Finland; ^5^Institute of Public Health and Clinical Nutrition, University of Eastern Finland, Kuopio, Finland; ^6^School of Medicine, University of Eastern Finland, Kuopio, Finland; ^7^Department of Pediatrics, Mikkeli Central Hospital, Mikkeli, Finland; ^8^Department of Pediatrics, Kuopio University Hospital, Kuopio, Finland

**Keywords:** childhood obesity, specialist care, cardiometabolic, treatment outcomes, blood pressure, fatty liver, metabolism, BMI SDS

## Abstract

**Background:** Childhood obesity exposes individuals to cardiometabolic disturbances. We analyzed how family-based multidisciplinary obesity treatment influenced children's cardiometabolic health.

**Materials and methods:** In this retrospective, two-year, follow-up study of 654 2- to 18-year-old children treated for obesity in three Finnish pediatric clinics in 2005–2012, blood pressure (BP), metabolic parameters, and the influence of sex, puberty and a change in body mass index standard deviation score (BMI SDS) were analyzed.

**Results:** At baseline, at least one cardiovascular risk factor was present in 474 (80%) cases. Boys presented with more significant changes in cardiometabolic parameters than girls during the treatment. Boys' total cholesterol (TC) improved by 12 months *(P* = 0.009), and their low-density lipoprotein C (LDL-C) and glycosylated hemoglobin ameliorated by 12 months (*P* = 0.030 and 0.022, respectively) and 24 months (*P* = 0.043 and 0.025, respectively). Boys' triglycerides, insulin, homeostasis model assessment for insulin resistance (HOMA-IR) and systolic BP deteriorated at 24 months (*P* < 0.001, 0.004, 0.002, and 0.037, respectively). In all children, the number of acceptable TC, LDL-C, insulin, and HOMA-IR values increased if BMI SDS reduced 0.25 or more by 12 months.

**Conclusion:** Minor cardiometabolic improvements were found during the obesity treatment. These findings indicate the need to assess treatment methods and focus on prevention.

## Introduction

Childhood obesity increases morbidity and premature mortality (1, 2). A wide spectrum of physical symptoms, psychosocial disturbances, and cardiovascular (CV) risk factors, even diseases, are related to childhood obesity (3–6). Moreover, childhood obesity tracks easily into adulthood (7). Due to the strong correlation between childhood and adulthood obesity, it is difficult to determine the independent effects of childhood obesity (8).

The processes of atherosclerosis and obesity-related complications already begin in childhood. Left ventricle hypertrophy and dysfunction have been documented in children with severe obesity (4, 9, 10). The components of the metabolic syndrome, such as dyslipidemia, hyperinsulinism, and hypertension, are common in adolescents with obesity (11). Moreover, fatty liver is a frequent consequence of childhood obesity (9, 12). As the disease processes begin in childhood, prevention and treatment for obesity should start early. It has been established that the risks for CV diseases and type 2 diabetes significantly decrease if an obese child does not become an obese adult (6, 11).

In randomized controlled clinical trials, the cardiometabolic short-term outcomes of childhood obesity treatment are moderately efficient, but the data on the long-term outcomes of these trials and on the outcomes of studies conducted in everyday clinical practice are insufficient (13–17). There is an urgent need to analyze efficacy of obesity treatments and to find better intervention methods, which could be implicated in clinical practice.

The purpose of this study was to analyze the cardiometabolic outcomes of a family-based multidisciplinary behavioral treatment of up to 2 years in length for childhood obesity in three Finnish pediatric units, to examine the influence of sex and puberty on the outcomes, and to explore the influence of body mass index standard deviation score (BMI SDS) change during treatment on the metabolic profile.

## Materials and methods

### Sample and study design

This is a retrospective, register-based longitudinal study of 654 children aged 2 to 18 years treated for obesity in the period 2005–2012 in three pediatric units of Eastern Finland (Kuopio University Hospital, Mikkeli Central Hospital, or North Karelia Central Hospital). These hospitals are responsible for pediatric secondary and tertiary care in their hospital districts. The participants in this study were included in our first report on children (*n* = 900) evaluated for obesity and cardiometabolic profile at the time of the baseline visit in specialist care (18) and in our second report (*n* = 654) on the BMI SDS outcomes of the obesity treatment (19). The children in the present study had to have one or more follow-up visits with a pediatrician during the study period. The data were analyzed at three different time points: at baseline (the first visit), 12 months (6–17.9 months, *n* = 521) from baseline, and 24 months (18–30 months, *n* = 345) from baseline. The included and excluded children did not differ in terms of sex {Mann-Whitney U [M-W U], *P* = 0.631}, age (M-W U, *P* = 0.087), puberty {Pearson chi-square [χ^2^], *P* = 0.375}, or BMI SDS (M-W U, *P* = 0.844) at the time of their baseline visit.

The treatment, described in detail in our previous report, was carried out by a multidisciplinary team, which consisted of a pediatrician, specialist nurse, dietician, physiotherapist, psychologist, and family therapist (19). The treatment was planned individually with each family and child according to the regional obesity treatment programs based on the Finnish National Current Care Guidelines on Childhood Obesity (20). Parental involvement, motivation, and long-term adherence to the protocol were important components of the treatment. Ambulatory treatment lasted approximately a year, but children with severe obesity and children who already had significant metabolic disturbances remained in specialist care for a longer time.

The Research Ethics Committee of the Hospital District of Northern Savo (Kuopio, Finland) has approved the study protocol. Permission to use the patient registers was obtained from the National Institute for Health and Welfare and from the participating hospitals.

### Assessments

The children's height and weight were measured and recorded on visits by an experienced nurse. Height was measured by using a wall-mounted Harpenden stadiometer (Holtain Ltd, Crymyck, UK) with an accuracy of 0.1 cm. The mean of the closest two out of three measurements of barefoot height was used. Weight was measured in light underwear using a calibrated electronic scale with an accuracy of 0.1 kg as the mean of two measurements. BMI was calculated as weight divided by height squared. Height and BMI values were converted into body height SDS and BMI SDS according to Finnish gender- and age-specific population standards (21).

The children were classified at baseline into the following age groups: 2–6.9 years (13%), 7–9.9 years (20%), 10–14.9 years (53%), and 15–18 years (14%). Children who were 10 years old or older were considered adolescents (22). Pubertal status was recorded by physicians using the Tanner staging method (23, 24). For the purpose of this study, children were classified as prepubertal (45%) and pubertal (55%) at baseline and as prepubertal (25%) and pubertal (75%) over the 2-year follow-up. Girls with palpable breast tissue and boys with testicular volume >3 ml were designated as pubertal (25). BMI SDS was used to classify children into four obesity categories: overweight, obesity, severe obesity, and morbid obesity. According to Finnish growth standards the BMI SDS cut-offs for each category for girls were 1.16, 2.11, 2.76, and 3.24, respectively, and for boys 0.78, 1.70, 2.36, and 2.85, respectively. These values corresponded to BMIs of 25, 30, 35, and 40 kg/m^2^, respectively, at the age of 18 years (21). At baseline, 9% of the children were overweight, 47% had obesity, 31% had severe obesity, and 13% had morbid obesity. Using the cut-offs of the International Obesity Task Force, 6% of the children were overweight and 94% obese (26). The BMI SDS changes from baseline were defined at 12 and 24 months. Study subjects were categorized at 12 months in three groups according to the BMI SDS reduction from baseline: BMI SDS reduction ≥ 0.25 units (good), 0–0.24 units (borderline), and no reduction (poor). BMI SDS reduction ≥ 0.25 has been reported as necessary to improve CV risk factors in overweight children (27).

### Blood pressure

Blood pressure (BP) was measured two or three times using the Criticon Dinamap Vital Signs monitor 1846 SX with a suitable Duracuff (8–13 or 38–50 cm) from the right arm in the supine position after a recommended 15 min of rest while seated, and the lowest recording was registered. Systolic BP (SBP) and diastolic BP (DBP) were classified at baseline as normal, high normal, and hypertensive (stages 1 and 2). The height percentile-, age-, and gender-specific percentile cut-offs were used (<90th, 90th but less than 95th and ≥ 95th), as recommended by the fourth report from the National High Blood Pressure Education Program (NHBPEP) Working Group on Children and Adolescents (28). For clinical purposes, the hypertensive category was divided into two subgroups: stage 1 hypertension (BP between the 95th percentile and the 99th percentile plus 5 mmHg) and stage 2 hypertension (BP above the 99th percentile plus 5 mmHg).

### Laboratory analyses

The laboratory analyses done in the 6-month periods before and after the first visit, 12-month visit, and 24-month visit were included. All samples were taken after a recommended 12-h overnight fast. Each hospital carried out the analyses in their own laboratories in 2005–2007. From 2008, all laboratory analyses were performed in one regional laboratory, the Eastern Finland Laboratory Center. We calculated possible differences between analyses carried out before and after the move to central laboratory analysis using separate general linear models for boys and girls, controlling for age, pubertal status and obesity status.

Total plasma cholesterol (P-TC) and plasma triglyceride (P-TG) were analyzed with a colorimetric enzymatic assay, and plasma low-density lipoprotein cholesterol (P-LDL-C) and plasma high-density lipoprotein cholesterol (P-HDL-C) were analyzed with a homogeneous colorimetric enzymatic assay (both Roche Diagnostics GmbH, Mannheim, Germany). The IFCC kinetic method was used to quantify plasma alanine aminotransferase (P-ALT) (Roche Diagnostics GmbH, Mannheim, Germany). Plasma glucose (P-Gluc) was analyzed by the hexokinase method, and blood glycosylated hemoglobin (B-HbA1c) was analyzed with a turbidimetric inhibition immunoassay (both Roche Diagnostics GmbH, Mannheim, Germany). Serum insulin (S-INS) was analyzed using an electrochemiluminescence immunoassay (Roche Diagnostics GmbH, Mannheim, Germany).

Fasting (f) P-Gluc concentrations were classified according to the International Society for Pediatric and Adolescent Diabetes (ISPAD) Clinical Practice Consensus Guidelines 2014 Compendium (29) as normal, impaired fP-Gluc (IFG), or diabetic: <5.6 mmol/l, 5.6–6.9 mmol/l, and ≥ 7 mmol/l, respectively. A dichotomous classification of fP-Gluc of normal (< 5.6 mmol/l) and abnormal (≥ 5.6 mmol/l) was used in McNemar's test. P-Gluc values following a 2-h oral glucose tolerance test (OGTT; a load of 1.75 g/kg anhydrous glucose up to a maximum of 75 g, dissolved in water) were classified as normal (< 7.8 mmol/l), impaired glucose tolerance (IGT) (7.8–11.0 mmol/l), or diabetic (≥ 11.1 mmol/l). B-HbA1c% was classified as normal (< 5.8%), prediabetic (5.8–6.4%), or diabetic (≥ 6.5%). Prediabetes was recognized in cases where there was IFG or IGT, or HbA1c in the prediabetic range (29). Fasting S-INS concentrations were categorized as normal or hyperinsulinemic (HI) using the following pubertal stage-specific cut-offs for hyperinsulinemia: prepubertal, >15 mU/l; pubertal, >30 mU/l; and postpubertal, >20 mU/l (30). The homeostasis model assessment for insulin resistance (HOMA-IR) was calculated by the formula fS-INS (mU/l) × fP-Gluc (mmol/l)/22.5. The cut-offs for normal and abnormal HOMA-IR values were 2.67 for prepubertal boys and 5.22 for pubertal boys, and 2.22 and 3.82 for prepubertal and pubertal girls, respectively (30).

Lipid levels were classified as acceptable, borderline, or high (or low for HDL-C), in accordance with the Summary Report (2011) of the Expert Panel on Integrated Guidelines for Cardiovascular Health and Risk Reduction in Children and Adolescents (28), as follows: fP-TC: < 4.40, 4.40–5.17, and > 5.18 mmol/l, respectively; fP-LDL-C: < 2.84, 2.84–3.35, and ≥3.36 mmol/l, respectively; fP-TG for children under 10 years old, < 0.84, 0.84–1.12, and ≥1.13 mmol/l, respectively; for adolescents, < 1.02, 1.02–1.46, and ≥1.47 mmol/l, respectively. The cut-offs of fP-HDL-C sub-groups were acceptable, borderline and low, defined as > 1.17, 1.04–1.17, and < 1.04 mmol/l, respectively. Fasting P-ALT data were classified as elevated at ≥ 40 IU/l (31).

CV risk factors comprised hypertensive SBP or DBP, high P-TC, high P-LDL-C, high P-TG, low P-HDL-C, and diabetes or prediabetes.

### Statistical analyses

Descriptive data were analyzed according to sex, and the data were either presented as the means and 95% confidence intervals (CIs) or as medians and interquartile ranges (IQR), according to the distribution of the data variables. The distributions for normality were tested with the Shapiro-Wilk test and visualized with the histograms. Metabolic parameters and BP were presented also in staged distributions. Continuous variables were compared using the independent samples *t*-test for normally distributed variables and the M-W U test for non-normally distributed variables. The Pearson χ^2^ test was used to compare distributions at one time point.

Because of the large variety in timing of the visits and repeated cardiometabolic measurements, cardiometabolic outcomes were analyzed using linear mixed model analysis. To obtain a better normality in the distributions of metabolic parameters, the log-transformed values were used and to avoid negative metabolic values, a constant value (one) was added to all metabolic values before the logarithms were calculated. To analyze whether the study subjects benefitted from treatment, cardiometabolic measurements at baseline were compared with those over the entire treatment period using linear mixed model analyses. Consequently, three time points were categorized dichotomously (baseline group and 12 and 24 months together as another group). Continuous cardiometabolic parameters were introduced one by one in the model as a dependent variable, time points as fixed effects, and study subjects as random effects. Because age and BMI SDS could influence the levels of dependent variables, all analyses were adjusted for age and BMI SDS at baseline including these covariates as fixed effects to the models. Linear mixed model analyses were conducted for all study subjects and separately for girls and boys. Moreover, all the analyses were conducted for all prepubertal and pubertal children and separately for prepubertal and pubertal girls and boys. The results were represented as the mean differences and 95% CIs. Residuals' normality was used to evaluate the validity of the assumptions of the used mixed models.

Furthermore, to analyze cardiometabolic changes at time points, the comparisons between 12 months and baseline and between 24 months and baseline were performed using linear mixed model analysis as described above with an exception of time points. In these comparisons, all three time points (baseline, 12 and 24 months) were included in the models as fixed effects. The analyses were conducted for all study subjects and separately for girls and boys. The mean differences between time points were represented using the traffic light method where the green color indicated improvement and the red color deterioration of the cardiometabolic parameter.

The influence of the change in BMI SDS on the changes of metabolic parameters was studied using categorized variables and the McNemar's test. The categorized metabolic parameters (acceptable, borderline, and high/low or normal and abnormal) at baseline were compared to their pairs at 12 months in three subgroups of BMI SDS change at 12 months from baseline (good, borderline, and poor). These comparisons of metabolic distributions were done using both authentic paired data and data created by the intention-to-treat approach to avoid the problem of missing measurements (data not shown). The influence of BMI SDS change was analyzed only at 12 months. At 24 months, the number of cases in some subgroups was too small for reliable comparisons. The results were represented in bar graphs.

Statistical analyses were performed using SPSS 21.0, (IBM Corporation, New York, USA). A *p*-value of <0.05 was considered statistically significant.

## Results

Table 1 describes the background clinical characteristics of study subjects. Of the 654 children (53% boys) at baseline, 68% were adolescents and 55% were pubertal. Over the entire follow-up, 463 (75%) study subjects were pubertal. The median (IQR) age was 11.9 (9.2, 14.1) years, and the mean (95% CI) BMI SDS was 2.52 (2.48, 2.56). There were significantly more boys than girls among subjects with severe and morbid obesity at baseline and at both 12 and 24 months (Tables 1, 2). There was no sex difference in BMI SDS reduction and BMI SDS decreased by at least 0.25 in 24% of children both from baseline to 12 months and to 24 months as reported in our previous study (19).

**Table 1:** Background characteristics of the study subjects.

	**Girls *n* (%)**	**Boys *n* (%)**	***P***
All *n* (%)	302 (46.2)	352 (53.8)	
Age^a^, years	11.6 (8.9, 14.1)	12.0 (9.3, 13.9)	0.580^d^
**AGE, YEARS**			
2–6.9	43 (14.2)	38 (10.8)	0.204^e^
7–9.9	63 (20.9)	66 (18.8)	
10–14.9	149 (49.3)	202 (57.3)	
15–18	47 (15.6)	46 (13.1)	
Adolescence^b^			
Children	106 (35.1)	104 (29.6)	0.129^e^
Adolescents	196 (64.9)	248 (70.4)	
Pubertal stage^g^ recorded	299	347	
Prepubertal	99 (33.1)	189 (54.5)	<0.001^e^
Pubertal	200 (66.9)	158 (45.5)	
**BMI SDS**^c^^,^^h^	2.59 (2.53, 2.64)	2.45 (2.40, 2.51)	
Obesity stage^d^ *n*	302	352	
Overweight	43 (14.2)	15 (4.3)	<0.001^e^
Obesity	164 (54.3)	143 (40.6)	
Severe obesity	69 (22.8)	134 (38.1)	
Morbid obesity	26 (6.7)	60 (17.0)	
Obesity (IOTF)^e^ *n*	302	352	
Overweight	17 (5.6)	20 (5.7)	0.977^e^
Obesity	285 (94.4)	332 (94.3)	
Height SDS^c^^,^^h^	0.5 (0.4, 0.7)	0.5 (0.3, 0.6)	
SBP and DBP *n*	242	281	
SBP^c^^,^^i^, mmHg	121 (119, 123)	123 (121, 125)	
Normal	88 (36.4)	110 (39.1)	0.689^e^
High normal	27 (11.1)	26 (9.3)	
Stage 1	91 (37.6)	97 (34.5)	
Stage 2	36 (14.9)	48 (17.1)	
DBP^c^^,^^i^, mmHg	70 (69, 72)	70 (69, 71)	
Normal	158 (65.2)	207 (73.6)	0.206^e^
High normal	43 (17.8)	35 (12.5)	
Stage 1	37 (15.3)	35 (12.5)	
Stage 2	4 (1.7)	4 (1.4)	
Acanthosis nigricans	225	249	
Yes	139 (61.8)	157 (63.1)	0.775^e^
No	86 (38.2)	92 (36.9)	

*Results for girls and boys are represented as n (%) for distributions*,

and as

a median and interquartile ranges (IQR25,75) for not normally distributed age and as

c *means and 95% confidence intervals (CI) for normally distributed continuous variables*.

d *Mann-Whitney test*;

e *Pearson's chi-square test*.

A P-value < 0.05 is statistically significant. Categorized on the basis of

b *the definition by WHO (22)*,

g *the Tanner method (23, 24)*,

h *the Finnish growth references (21)*,

and n the

i *NHBPEP Working Group on Children and Adolescents; Stages 1 and 2 are hypertensive (28). BMI SDS, body mass index standard deviation score; IOTF, International Obesity Task Force; SBP, systolic blood pressure; DBP, diastolic blood pressure; NHBPEP, The National High Blood Pressure Education Program*.

**Table 2:** BMI SDS, changes in BMI SDS from baseline, and obesity stage distributions at 12 and 24 months from baseline in girls and boys.

	**12 months (*****n*** = **521)**			**24 months (*****n*** = **345)**		
	**Girls**	**Boys**	***P***	**Girls**	**Boys**	***P***
*n* (%)	243 (46.6)	278 (53.4)		157 (45.6)	188 (54.4)	
BMI SDS^a^^,^^b^	2.48 (2.42, 2.55)	2.38 (2.32, 2.43)		2.50 (2.44, 2.56)	2.38 (2.32, 2.44)	
Change in BMI SDS from baseline^c^	−0.10 (−0.14, −0.06)	−0.09 (−0.12, −0.05)		−0.09 (−0.13, −0.04)	−0.08 (−0.04, 0.05)	
**REDUCTION IN BMI SDS**						
≥0.25 (good)	63 (25.9)	60 (21.6)	0.144^d^	42 (26.8)	41 (21.8)	0.297^d^
0.24 to 0 (borderline)	83 (34.2)	118 (42.4)		43 (27.4)	65 (34.6)	
<0 (poor)	97 (39.9)	100 (36.0)		72 (45.8)	82 (43.6)	
**OBESITY STAGE**^b^						
Normal weight	2 (0.8)	1 (0.4)	<0.001^d^	2 (1.3)	0	0.007^d^
Overweight	46 (18.9)	15 (5.4)		23 (14.6)	16 (8.5)	
Obesity	125 (51.5)	117 (42.1)		79 (50.3)	74 (39.4)	
Severe obesity	58 (23.9)	105 (37.7)		40 (25.5)	70 (37.2)	
Morbid obesity	12 (4.9)	40 (14.4)		13 (8.3)	28 (14.9)	

a *BMI SDS is normally distributed and represented as means and 95% CIs*.

b *Based on the Finnish growth references (21)*.

*Distributions are represented as n (%)*.

c Revealed in linear mixed model analysis adjusted for age at baseline, expressed as mean difference (95% CI) from baseline, a negative value signifies a reduction, P for all comparisons was < 0.001 (19)

d *Pearson's chi-square test for differences between boys and girls. A P value > 0.05 is statistically significant. BMI SDS, body mass index standard deviation score; CI, confidence interval*.

To describe the cardiometabolic status of study subjects over the treatment, BP measurements and metabolic measurements and their distributions into acceptable, borderline, and high or low categories are presented in Table 3. Boys had more elevated P-ALT than girls at baseline, at 12 and 24 months (χ^2^, 0.003, 0.019, and 0.003, respectively) and more IFG at baseline and at 24 months (χ^2^, 0.048, and 0.029, respectively). Elevated P-ALT at baseline was associated with acanthosis nigricans (χ^2^, *P* = 0.003) and moreover, it was more frequent in children whose HOMA-IR indicated insulin resistance than in children whose HOMA-IR was normal (32 vs. 20%; χ^2^, *P* = 0.035). At baseline, HOMA-IR was non-acceptable in 108 (58%) boys and in 138 (83%) girls (χ^2^, *P* < 0.001), and it was significantly related to acanthosis nigricans (χ^2^, *P* < 0.001). There were no significant sex differences in the distributions of lipids or in the P-LDL-C to P-HDL-C and the P-TG to P-HDL-C ratios at any time point.

**Table 3:** Metabolic and blood pressure measurements in girls and boys at baseline, at 12 and 24 months.

	**Girls** ***n*** **(%)^a^**			**Boys** ***n*** **(%)^a^**		
	**Baseline**	**12 months**	**24 months**	**Baseline**	**12 months**	**24 months**
fP-ALT^b^, *n*	177	88	60	227	94	82
IU/l	24 (18, 35)	23 (17, 35)	21 (15, 35)	29 (29, 47)	33 (22, 61)	34 (25, 55)
< 40	144 (81.4)	70 (79.5)	48 (80.0)	155 (68.3)	60 (63.8)	46 (56.1)
>40	33 (18.6)	18 (20.5)	12 (20.0)	72 (31.7)	34 (36.2)	36(43.9)
fP-TC^c^, *n*	222	113	79	247	102	92
mmol/l	4.45 (3.90, 5.10)	4.50 (3.80, 5.10)	4.60 (4.10, 5.10)	4.50 (3.70, 5.10)	4.50 (4.00, 5.20)	4.45 (3.80, 5.00)
Acceptable	100 (45.0)	51 (45.1)	28 (35.4)	114 (46.2)	42 (41.2)	43 (46.7)
Borderline	70 (31.5)	37 (32.6)	33 (41.8)	75 (30.4)	33 (32.4)	32 (34.8)
High	52 (23.5)	25 (22.3)	18 (22.8)	58 (23.4)	27 (26.4)	17 (18.5)
fP-LDL-C^c^, *n*	212	112	79	233	99	92
mmol/l	2.80 (2.30, 3.38)	2.75 (2.20, 3.36)	2.90 (82.23, 3.38)	2.80 (2.28, 3.30)	2.85 (2.40, 3.40)	2.70 (2.23, 3.38)
Acceptable	121 (57.1)	63 (56.2)	37 (46.8)	126 (54.1)	49 (49.5)	52 (56.5)
Borderline	38 (17.9)	21 (18.8)	21 (26.6)	55 (23.6)	24 (24.2)	17 (18.5)
High	53 (25.0)	28 (25.0)	21 (26.6)	52 (22.3)	26 (26.3)	23 (25.0)
fP-HDL-C^c^, *n*	216	112	79	244	100	93
mmol/l	1.13 (0.97, 1.33)	1.12 (0.99, 1.33)	1.11 (0.92, 1.28)	1.12 (0.95, 1.35)	1.06 (0.89, 1.33)	1.05 (0.88, 1.32)
Acceptable	97 (44.9)	45 (40.2)	30 (38.0)	100 (41.0)	39 (39.0)	37 (39.7)
Borderline	51 (23.6)	31 (27.7)	20 (25.3)	53 (21.7)	19 (19.0)	18 (19.4)
Low	68 (31.5)	36 (32.1)	29 (36.7)	91 (37.3)	42 (42.0)	38 (40.9)
fP-TG^c^, *n*	219	110	78	245	97	88
mmol/l	1.21 (0.90, 1.68)	1.19 (0.81, 1.68)	1.19 (0.91, 1.76)	1.09 (0.77, 1.57)	1.25 (0.83, 1.65)	1.27 (0.82, 1.82)
Acceptable	65 (29.7)	37 (33.6)	25 (32.1)	101 (41.2)	32 (33.0)	30 (34.1)
Borderline	67 (30.6)	31 (28.2)	24 (30.8)	59 (24.1)	25 (25.8)	21 (23.9)
High	87 (39.7)	42 (38.2)	29 (37.1)	85 (34.7)	40 (41.2)	37 (42.0)
fP-Gluc^d^, *n*	222	124	84	249	111	93
mmol/l	5.30 (5.10, 5.70)	5.40 (5.10, 5.68)	5.30 (5.10, 5.60)	5.50 (5.20, 5.80)	5.50 (5.20, 5.80)	5.50 (5.20, 5.90)
< 5.6	152 (68.5)	82 (66.1)	57 (67.9)	145 (58.2)	65 (58.6)	47 (50.5)
5.6-6.9 (IFG)	67 (30.2)	39 (31.5)	26 (31.0)	102 (41.0)	46 (41.4)	46 (49.5)
> 7.0	3 (1.3)	3 (2.4)	1 (1.1)	2 (0.8)	0 (0.0)	0 (0.0)
2-h-OGTT-Gluc^d^, *n*	69	26	24	80	34	35
mmol/l	6.4 (5.50, 7.30)	5.50 (4.95, 6.10)	5.75 (5.30, 6.98)	6.35 (5.50, 7.30)	6.75 (5.78, 7.73)	6.70 (5.50, 7.60)
< 7.8,	57 (82.6)	21 (80.8)	21 (87.5)	66 (82.5)	26 (76.5)	27 (77.1)
7.8–11.0 (IGT)	10 (14.5)	4 (15.4)	3 (12.5)	13 (16.3)	8 (23.5)	8 (22.9)
>11.1	2 (2.9)	1 (3.8)	0	1 (1.2)	0	0
B-HbA1c^d^, *n*	80	57	38	81	66	42
mmol/l	35 (33, 38)	36 (33, 37)	36 (33, 37)	36 (34, 39)	36 (34, 38)	36 (33, 38)
< 5.7	73 (91.2)	51 (89.5)	35 (92.1)	66 (81.6)	57 (86.4)	36 (85.7)
5.8–6.4	4 (5.0)	4 (7.0)	3 (7.9)	12 (14.8)	8 (12.1)	6 (14.3)
>6.4	3 (3.8)	2 (3.5)	0	3 (3.6)	1 (1.5)	0
fS-INS^e^, *n*	168	105	69	191	88	61
mU/l	24 (16, 32)	24 (16, 34)	25 (18,36)	17 (11, 27)	23 (15, 34)	22 (16, 36)
Normal,	85 (50.6)	54 (51.4)	29 (42.0)	110 (57.6)	50 (56.8)	30 (49.2)
High	83 (49.4)	51 (48.6)	40 (58.0)	81 (42.4)	38 (43.2)	31 (39.8)
HOMA-IR^f^^,^^g^	6.0 (4.0, 8.1)	5.6 (3.6, 8.2)	6.6 (4.4, 8.6)	4.1 (2.7, 7.0)	5.5 (3.5, 8.4)	5.3 (4.0, 8.7)
Acceptable	27 (16.4)	21 (21.4)	8 (12.1)	76 (41.3)	31 (36.9)	21 (35.6)
High	138 (83.6)	77 (78.6)	58 (87.9)	108 (58.7)	53 (63.1)	38 (64.4)
BP, *n*	243	171	134	279	188	161
SBP^h^, mmHg	121 (119, 123)	120 (119, 122)	122 (120, 125)	123 (121, 125)	124 (122, 126)	124 (122, 126)
DBP^h^, mmHg	70 (69, 72)	70 (69, 72)	70 (68, 72)	70 (69, 71)	70 (69, 72)	70 (69, 72)

a *All continuous metabolic parameters are non-normally distributed and represented as medians and interquartile ranges (IQR25,75)*.

h *Normally distributed SBP and DBP are represented as means and 95% confidence intervals (CIs) for girls and boys at baseline, at 12 and 24 months from baseline. Categorized parameters and their distributions are represented as n (%)*.

b *Classified as elevated > 40 IU/l (31)*.

c *Categorized on the basis of NCEP (28)*.

d *fP-Gluc, IFG, 2-h-OGTT, IGT, and B-HbA1c classified on the basis of ISPAD (29)*.

e *High: > 15 mU/l in prepuberty, > 30 mU/l in puberty, and > 20 mU/l in postpuberty (30)*.

f *Calculated using the formula: fS-INS (mU/l) x fP-Gluc (mmol/l)/22.5 (30)*.

g *High: > 2.67 in prepubertal boys and > 5.22 in pubertal boys, and in girls > 2.22 and > 3.82, respectively (30). fP-ALT, fasting plasma alanine transferase; fP-TC, fasting plasma total cholesterol; fP-LDL-C, fasting plasma low-density lipoprotein cholesterol; fP-HDL-C, fasting plasma high-density lipoprotein cholesterol; TG, triglyceride; fP-Gluc, fasting plasma glucose; 2-h-OGTT-Gluc, 2-h oral glucose tolerance test; B-HbA1c, blood glycosylated hemoglobin; fS-INS, fasting serum insulin; HOMA-IR, homeostasis model assessment for insulin resistance; SBP, systolic blood pressure; DPP, diastolic blood pressure; NCEP, National Cholesterol Education Program Expert Panel; ISPAD, International Society for Pediatric and Adolescent Diabetes*.

The changes in metabolic parameters and BP from baseline throughout the entire two-year follow-up revealed in linear mixed model analyses adjusted for age and BMI-SDS at baseline are presented in Table 4. In all children, P-TC and P-LDL-C levels and B-HbA1c% values decreased significantly. In boys, P-TC, P-LDL-C, and P-HDL-C levels decreased (*P* = 0.008, 0.008, and 0.029, respectively), and P-TG levels increased (*P* = 0.008). Moreover, in boys, B-HbA1c% decreased, but S-INS levels and HOMA-IR values increased (*P* = 0.007, 0.024, and 0.011, respectively). In P-Gluc or P-ALT levels, no significant changes were detected throughout follow-up. Neither SBP nor DBP changed significantly. Boys presented with more significant changes in cardiometabolic parameters than girls during the treatment. Table 4 also presents the effect of puberty on cardiometabolic changes. The increase in S-INS levels and HOMA-IR was detected in both sexes only in prepuberty. Girls' P-TC and P-LDL levels decreased in prepuberty but in boys, they decreased in puberty. P-TG levels decreased in pubertal girls but increased in pubertal boys. Puberty did not influence BP levels over the entire treatment.

**Table 4:** Comparisons between cardiometabolic measurements during two-year follow-up and at baseline revealed in linear mixed model analyses adjusted for age and BMI SDS at baseline in all study subjects, girls, boys, and in prepubertal and pubertal children.

	**All^a^**			**Girls^a^**			**Boys^a^**		
	**Mean difference**	**95% CI for mean difference**	***P***	**Mean difference**	**95% CI for mean difference**	***P***	**Mean difference**	**95% CI for mean difference**	***P***
fP-ALT, IU/L	−0.007	−0.033, 0.018	0.570	−0.038	0.077, 0.001	0.055	0.019	−0.015, 0.052	0.272
Prepubertal	−0.015	−0.057, 0.027	0.478	−0.330	−0.109, 0.042	0.369	−0.005	−0.057, 0.047	0.859
pubertal	−0.005	−0.037, 0.027	0.756	−0.037	−0.082, 0.009	0.110	0.028	−0.015, 0.071	0.205
fP-TC, mmol/l	−0.010	−0.016, −0.004	**0.002**	−0.008	−0.017, 0.000	0.061	−0.011	−0.019, −0.003	**0.008**
Prepubertal	−0.015	−0.026, −0.004	**0.011**	−0.031	−0.050, −0.011	**0.003**	−0.006	−0.020, 0.008	0.404
Pubertal	−0.007	−0.014, 0.000	0.052	−0.003	−0.013, 0.007	0.573	−0.013	−0.023, −0.002	**0.019**
fP-LDL-C, mmol/l	−0.011	−0.019, −0.003	**0.005**	−0.008	−0.019, 0.003	0.171	−0.015	−0.026, −0.004	**0.008**
Prepubertal	−0.016	−0.031, −0.001	**0.032**	−0.039	−0.065, −0.013	**0.005**	−0.004	−0.021, 0.014	0.670
Pubertal	−0.009	−0.018, 0.000	0.063	−0.001	−0.013, 0.012	0.927	−0.019	−0.034, 0.005	**0.009**
fP-HDL-C, mmol/l	−0.004	−0.009, 0.001	0.096	−0.001	−0.008, 0.006	0.823	−0.007	−0.014, −0.001	**0.029**
Prepubertal	−0.006	−0.016, 0.004	0.226	−0.010	−0.030, 0.011	0.341	−0.003	−0.014, 0.007	0.522
Pubertal	−0.003	−0.008, 0.003	0.324	0.002	−0.006, 0.009	0.635	−0.008	−0.017, 0.000	0.060
fP-TG, mmol/l	0.006	−0.006, 0.019	0.342	−0.012	−0.030, 0.006	0.193	0.024	0.006, 0.041	**0.008**
Prepubertal	0.018	−0.003, 0.040	0.097	0.031	−0.004, 0.066	0.080	0.011	−0.018, 0.040	0.444
Pubertal	0.001	−0.014, 0.016	0.902	−0.021	−0.042, −0.001	**0.043**	0.027	0.005, 0.050	**0.017**
fP-Gluc, mmol/l	−0.001	−0.005, 0.002	0.491	−0.002	−0.006, 0.003	0.523	0.000	−0.005, 0.004	0.887
Prepubertal	0.002	−0.006, 0.010	0.555	0.014	0.004, 0.023	**0.010**	−0.004	−0.014, 0.006	0.458
Pubertal	−0.002	−0.006, 0.002	0.319	−0.004	−0.009, 0.001	0.123	0.001	−0.004, 0.007	0.610
fS-INS, mU/l	0.023	−0.004, 0.050	0.089	−0.001	−0.037, 0.036	0.975	0.046	0.006, 0.085	**0.024**
Prepubertal	0.123	0.067, 0.179	<**0.001**	0.158	0.068, 0.249	**0.002**	0.105	0.033, 0.176	**0.005**
Pubertal	−0.003	−0.33, 0.027	0.864	−0.020	−0.058, 0.018	0.298	0.022	−0.027, 0.070	0.385
HOMA-IRb	0.023	−0.003, 0.049	0.078	−0.002	−0.037, 0.033	0.912	0.051	0.012, 0.090	**0.011**
Prepubertal	0.113	0.064, 0.162	<**0.001**	0.152	0.073, 0.232	**0.001**	0.096	0.035, 0.158	**0.003**
Pubertal	−0.001	−0.031, 0.028	0.921	−0.021	−0.057, 0.015	0.250	0.028	0.020, 0.077	0.247
B-HbA1c, mmol/l	−0.010	−0.019, −0.002	**0.020**	−0.001	−0.008, 0.005	0.719	−0.010	−0.017, −0.003	**0.007**
Prepubertal	−0.024	−0.056, 0.007	0.131	NAc	NAc	NAc	−0.034	−0.075, 0.006	0.097
Pubertal	−0.007	−0.015, 0.002	0.111	−0.001	−0.013, 0.011	0.852	−0.012	−0.022, −0.002	**0.018**
SBP, mmHg	0.569	−0.637, 1.776	0.354	−0.082	−1.828, 1.663	0.926	1.391	−0.267, 3.049	0.100
Prepubertal	0.805	−1.366, 2.977	0.465	0.058	−3.108, 3.223	0.971	1.210	−1.729, 4.148	0.416
Pubertal	0.423	−1.043, 1.888	0.571	−0.026	−2.051, 1.999	0.980	1.347	−0.748, 3.441	0.207
DBP, mmHg	−0.217	−1.072, 0.639	0.619	−0.399	−1.708, 0.909	0.547	−0.038	−1.149, 1.091	0.959
Prepubertal	0.687	−0.999, 2.373	0.422	−0.113	−2.996, 2.770	0.938	1.432	−0.678, 3.542	0.181
Pubertal	−0.584	−1.599, 0.431	0.259	−0.456	−1.936, 1.024	0.545	−0.739	−2.123, 0.644	0.294

*Metabolic parameters were not normally distributed and to reach normal distributions they were log. transformed before analyses*.

a *The results are represented as mean difference from baseline and its 95% CI in all study subjects, girls, boys, and in prepubertal and pubertal children (23, 24). A negative mean difference expresses a reduction from baseline. A P value < 0.05 is statistically significant (in bold font). ^b^HOMA-IR was calculated using the formula: fS-INS (mU/l) × fP-Gluc (mmol/l)/22.5. ^c^The number of events was too small for reliable analyses. BMI SDS, body mass index standard deviation score; CI, confidence interval; fP-ALT, fasting plasma alanine transferase; fP-TC, fasting plasma total cholesterol; fP-LDL-C, fasting plasma low-density lipoprotein cholesterol; fP-HDL-C, fasting plasma high-density lipoprotein cholesterol; fP-TG, fasting plasma triglyceride; fP-Gluc, fasting plasma glucose; fS-INS, fasting serum insulin, HOMA-IR, homeostasis model assessment for insulin resistance; B-HbA1c, blood glycosylated hemoglobin; SBP, systolic blood pressure; DBP, diastolic blood pressure*.

**Figure 1:**
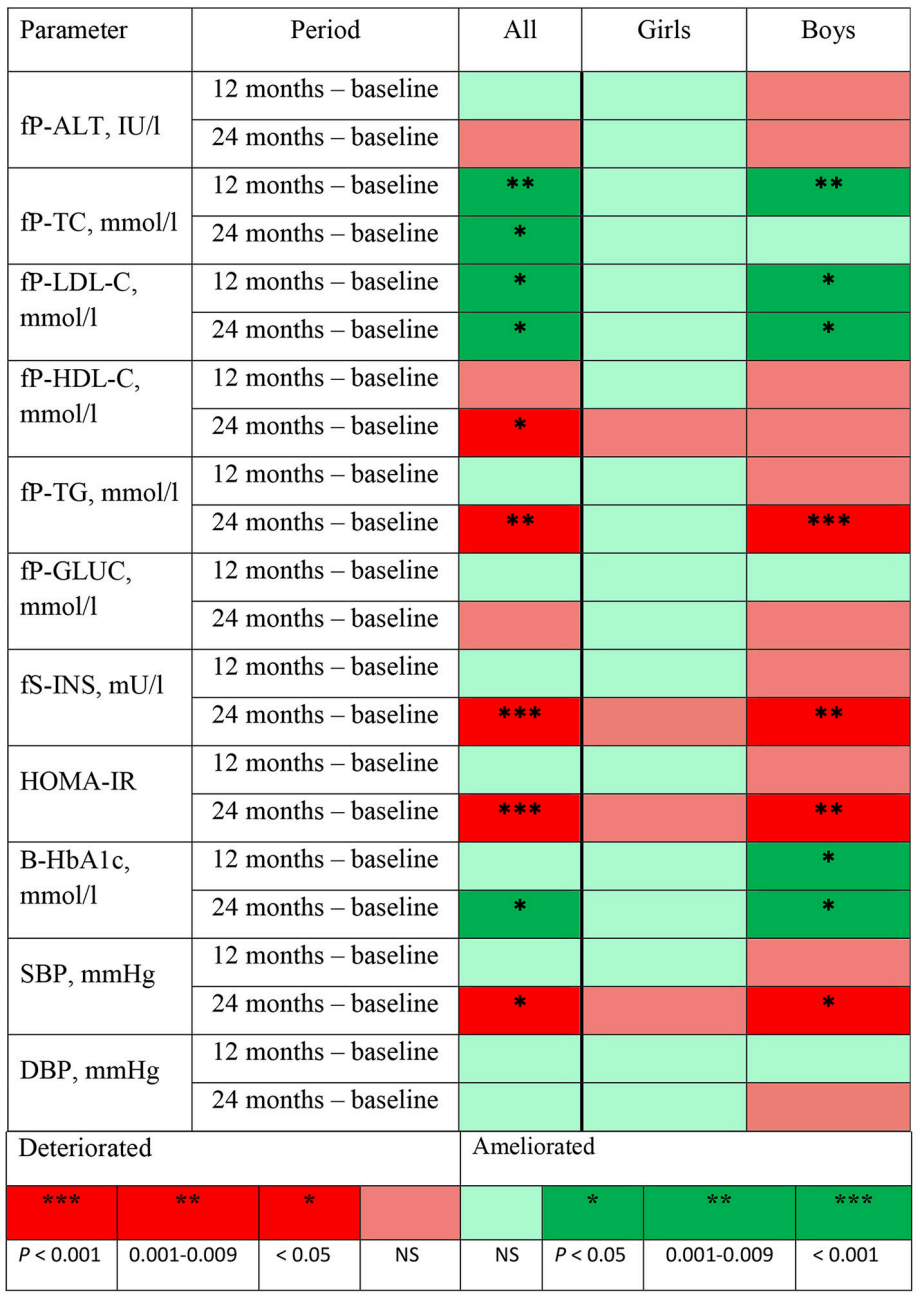
Changes in metabolic and BP measurements, their directions, and the significance of the change at 12 and 24 months from baseline revealed in mixed model analyses adjusted for age and body mass index standard deviation score at baseline presented using the traffic light method. fP-ALT, plasma alanine transferase; fP-TC, fasting plasma cholesterol; fP-LDL-C, fasting plasma low-density lipoprotein; fP-HDL-C, fasting plasma high-density lipoprotein; fP-TG, fasting plasma triglyceride; fP-Gluc, fasting plasma glucose; fS-INS, fasting serum insulin; HOMA-IR, homeostasis model assessment for insulin resistance calculated by using the formula fS-INS (mU/l) × fP-Gluc (mmol/l)/22.5; B-HbA1c, blood glycosylated hemoglobin; SBP, systolic blood pressure; DBP, diastolic blood pressure.

The changes in metabolic parameters and in BP at 12 and 24 months from baseline revealed in linear mixed model analyses are represented in Figure 1 using the traffic light method. In girls, there were no significant changes in any metabolic parameter or in BP at 12 or 24 months. In boys, a significant improvement (dark green) was detected at 12 months in P-TC levels (*P* = 0.009) and both at 12 and 24 months in P-LDL-C levels (*P* = 0.030 and 0.043, respectively) and in HbA1c% values (*P* = 0.013 and 0.025, respectively). Moreover, in boys, P-TG and S-INS levels and HOMA-IR values deteriorated (dark red) at 24 months from baseline (*P* < 0.001, 0.004, and 0.002, respectively). Boys' SBP levels increased significantly at 24 months (*P* = 0.037), but DBP levels remained unchanged both at 12 and 24 months.

**Figure 2:**
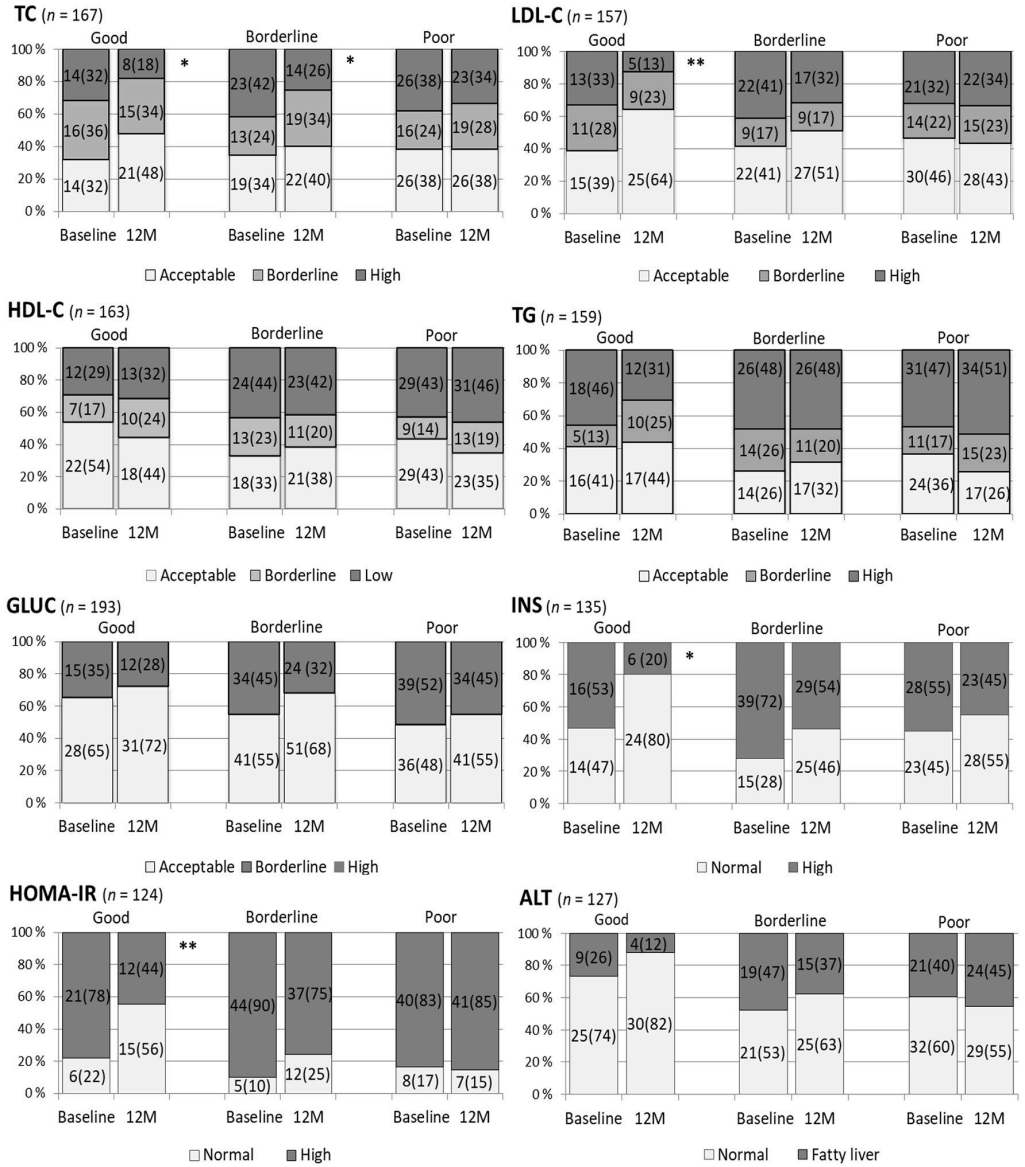
The influence of the staged change in body mass index standard deviation score (BMI SDS) on categorized metabolic variables at 12 months (M) compared to the situation at baseline. A BMI SDS reduction of 0.25 units or more from baseline was defined as good change, 0–0.24 borderline, and no reduction as poor. Plasma total cholesterol (TC) in mmol/l was categorized: acceptable < 4.40, high ≥ 5.18; plasma low-density lipoprotein cholesterol (LDL-C) in mmol/l, acceptable < 2.84, high ≥ 3.36; plasma high-density lipoprotein cholesterol (HDL-C) in mmol/l, acceptable > 1.17, low < 1.04; plasma triglyceride (TG) in mmol/l for < 10-year-olds, acceptable < 0.84, high ≥ 1.12 and for ≥ 10-year-olds, acceptable < 1.02 and high ≥ 1.47 (28). Plasma glucose (GLUC) cut-off for normal and elevated was 5.6 mmol/l (29). Serum insulin (INS) was classified as normal or high with cut-offs: in pre-puberty 15 mU/l, puberty 30 mU/l, and in postpuberty 20 mU/l (30). Homeostasis model assessment for insulin resistance (HOMA-IR) was calculated by the formula: fS-INS (mU/l) × fP-Gluc (mmol/l)/22.5 and its cut-offs for normal and high were 2.67 in prepubertal boys, 5.22 in pubertal boys and in girls 2.22 and 3.82, respectively (30). Plasma alanine transferase (ALT) values ≥ 40 IU/l expressed fatty liver (31). McNemar's test, **P 0.001–0.010, *P < 0.05.

The influence of BMI SDS changes on the categorized metabolic parameters at 12 months compared with those at baseline is represented in Figure 2. When the reduction in BMI SDS was poor, no significant favorable change in any metabolic profile was detected. Whereas when the BMI SDS reduction was good, 0.25 or more, the proportion of acceptable P-TC, P-LDL-C, S-INS, and HOMA-IR values were more prevalent at 12 months than at baseline (McNemar's test, *P* = 0.016, 0.007, 0.021, and 0.004, respectively). When the BMI SDS reduction was borderline (0–0.24), the acceptable proportions of P-Gluc, S-INS, HOMA-IR, and P-TG were augmented (*P* = 0.087, 0.052, 0.065, 0.063, respectively) but only the profile of TC was ameliorated significantly (*P* = 0.048). The comparison of the influence of BMI SDS change on the distributions of metabolic parameters at 12 months and at baseline was also done using the intention-to-treat method, and the results of this approach were very similar to the results presented in Figure 2.

## Discussion

In this study, the cardiometabolic outcome of the multidisciplinary family-based lifestyle treatment for childhood obesity presented some interesting findings. Boys presented with more significant changes in cardiometabolic parameters than girls during the treatment. In boys, P-TC, P-LDL-C, and HbA1c improved, but P-HDL-C, P-TG, S-INS, HOMA-IR deteriorated. The boys' significant deteriorations of TG, INS, HOMA-IR, and moreover, of SBP, were detected only at 24 months. P-Gluc, P-ALT, and DBP levels remained stable over the course of treatment both in girls and boys. At 12 months, for all children, the acceptable proportions of P-TC, P-LDL-C, S-INS, and HOMA-IR augmented significantly only if BMI SDS decreased at least 0.25 from baseline. On the other hand, even if there was no BMI SDS reduction at 12 months, no deterioration occurred in the distributions of lipids, Gluc, INS, HOMA-IR, or ALT.

Many cardiometabolic parameters change by age and puberty. Insulin resistance rises progressively from age 7 years, a few years before the first pubertal signs, and increases further at puberty (32). At puberty there is also a switch: prepubertal girls have higher insulin resistance but boys exhibit a higher metabolic risk by the end of puberty (33). These findings may partly explain the complexity of our results: S-INS and HOMA-IR increased in prepubertal girls and boys, despite of treatment. Surprisingly no change in these parameters was detected in pubertal children. This may be due to a low proportion of subjects with impaired glucose tolerance (14.5% in girls; 16.3% in boys) at baseline. Plasma lipids show significant physiological changes by age (34). P-TC and P-HDL-C reach their maximum levels by age 9 years and thereafter decline together with P-LDL-C. Unlike cholesterol levels, P-TG increases clearly in boys during puberty. In our study, P-TC and P-LDL-C decreased in prepubertal girls and pubertal boys. Whereas this prepubertal decrease in girls may well be due to lifestyle changes, the decrease in pubertal boys is not necessarily due to the treatment but may simply reflect a physiological change. The difference between the change of P-TG levels in pubertal boys and girls may also be due to a natural course of P-TG during puberty. The complexity of our findings highlights the importance of a proper control group, especially around puberty.

In our study, the prevalences of adverse levels of lipids were higher than those reported from Central Europe (35). In the meta-analysis published in 2013, lifestyle interventions led to significant improvements in P-LDL-C and P-TG up to one year from baseline, as well as improvements in P-HDL-C if the treatment included both dietary and exercise interventions (36). However, the results presented in different meta-analyses were not unequivocal. In a recent meta-analysis on childhood obesity interventions, no significant changes in lipids were reported (15). In recent prospective studies, a BMI SDS reduction of 0.25 or more was needed to improve P-TG and P-HDL-C significantly in a one-year lifestyle intervention (27, 37). In our study, TC and LDL-C were ameliorated, but HDL-C and TG deteriorated during the treatment even when the BMI SDS reduction was good. This finding is worrying, as the lipid levels in childhood are strongly correlated with the levels in adulthood, and this correlation, which is stronger in boys, was independent of the age at the time of measurements (38). Our results were also concerning because worsened P-HDL-C and P-TG are considered a part of the metabolic syndrome in all of its definitions.

Plasma glucose levels did not improve significantly in this study, although the high prevalence of IFG at baseline demanded its improvement. However, IFG was less frequent at 12 months than at baseline irrespective of the change in BMI SDS. Glucose levels did not improve in many other similar studies or meta-analyses (15, 36). Insulin resistance, defined by HOMA-IR, decreased at 12 months according to our results and this decrease was related to the degree of BMI SDS reduction (Figure 2). This finding is in line with those of many studies of insulin resistance in childhood (30, 39). Unfortunately, in our study, the levels of insulin and HOMA-IR in both sexes increased at 24 months, with a significant increase in boys. Better short than long term results in glucose metabolism could be due to more intensive lifestyle treatment during the first year of intervention. The boys' increased insulin resistance, together with the deteriorations of SBP and obesity lipids HDL-C and TG, evoke a concern of an early metabolic syndrome especially in boys (40).

Elevated P-ALT is considered a predictor of chronic nonalcoholic fatty liver disease (NAFLD). NAFLD is strongly related to visceral obesity, IR, dyslipidemia, and hypertension and is more frequent in boys. Moreover, in a recent meta-analysis, NAFLD was identified as a risk factor for subclinical abnormalities in the myocardium and in left ventricular function already in childhood (9). Many prospective cohort studies focusing on lifestyle treatment for NAFLD have reported a significant reduction in P-ALT related to a reduction in weight and IR (13, 41). However, poor compliance with the treatment worsened the outcomes (42). In accordance with other studies, we found that elevated P-ALT was associated with high HOMA-IR and acanthosis nigricans, but P-ALT did not ameliorate even if the reduction in BMI SDS was good and the lifestyle treatment included all the substantial elements. We can only speculate whether this finding was due to other concurrent metabolic disturbances, such as the high prevalence of IFG and elevated HOMA-IR, or perhaps the treatment time of 1 year was too short for liver tissue recovery.

Although there was a possibility of overestimation in the high prevalence of hypertensive SBP values (52% at baseline) because of an oscillometric measuring method and fear of measurements in children, the levels of BP remained high over the follow-up in comparison with other reports (3). Furthermore, SBP levels even significantly deteriorated in boys over the entire follow-up. In clinical practice, this finding indicates that there were many hypertensive children who should have been examined thoroughly to define the cause and the importance of high BP for assessing the need for antihypertensive medication. A 24-h ambulatory blood pressure monitoring should be performed in subject with elevated BP at rest, as it is the gold standard measure in children and adolescents. An important question is whether adequate attention is paid to hypertensive BP values in clinical practice. In other studies, variable BP outcomes in obesity treatment were described, and in most studies, SBP significantly decreased (36, 43).

Our study had some obvious limitations. It was a retrospective analysis of obesity treatment in clinical practice, and therefore, there was no standardized intervention protocol with a control group. A control group would have separated changes in cardiometabolic parameters caused by age and puberty from those caused by treatment. Because of individually programmed treatment, it was not possible to obtain data on all study subjects over the entire follow-up time. We could not know how well the participating families managed to carry out the advised lifestyle changes. Moreover, because there was no standardized protocol for laboratory analyses, these analyses were conducted only when necessary. This procedure might have caused a decrease in the normal range values rather than in the values out of reference range. The change in laboratory usage at the beginning of 2008 had a small effect on mean levels of P-HDL-C and P-Gluc in girls and on P-LDL-C in boys. It was, however, unlikely that these effects would have influenced the results. Finally, our study was conducted among obese children treated in specialist care, and therefore, the results may not be generalized to all overweight and obese children.

The concise strengths of this study were a relatively large number of study subjects, the cardiometabolic follow-up data up to 24 months, and the cardiometabolic outcome of childhood obesity treatment in real-life conditions. The data included all children who were treated in specialist care for obesity in the study region during the study period, and therefore, the results were representative of this group of patients. Furthermore, the study subjects were a homogenous group originating from Eastern Finland, and thus, children's cardiometabolic outcomes were not influenced by the difference in the ethnic background. Although this study was retrospective, the data up to the two-year follow-up were very reliably constructed because of the common treatment strategies in the participating clinics (20) and well-documented data and measurements. The growth data, for instance, were available in 100% of all clinical visits. In our study, the percentage of dropouts, namely, the number of those who left care without a recorded reason, was 3.6% of the original data. This is a very small percentage compared with other similar studies on childhood obesity. Finally, this is the first large study in Finland to describe the cardiometabolic outcomes of pediatric obesity treatment.

Against our expectations, there were only a few improvements in cardiometabolic status in this study. However, difficulty in achieving good cardiometabolic responses to treatment corresponded with findings from the recent meta-analyses done on the randomized controlled studies, which often had a very limited number of study subjects, varying treatment protocols, a notable number of dropouts, and usually no follow-up time at all (14–16). Our study supported previous statements of the need for at least a 0.25 reduction in BMI SDS (27, 37).

An interesting finding in our study was the different cardiometabolic response in boys and girls to the obesity treatment even after adjusting for age and although there was no significant sex difference in BMI SDS reduction over treatment time, and moreover, no significant sex difference in the adherence to the protocol (19). In girls, most metabolic parameters, except S-INS and HOMA-IR, which were already worse in girls than in boys at baseline, improved during the treatment, although not significantly. Most of the deteriorations in metabolism occurred in boys. Surely there is no simple explanation for the sex difference in metabolic outcomes. We can only speculate whether the girls already had more healthy diets and dietary habits before coming into care, and thus, the lifestyle changes were less effective for them, or if body composition was different in boys and girls and enabled the treatment responses. Were the boys perhaps less obedient to the lifestyle advice, or are the results pure reality and anticipation for males' increased risk for the early CV events and higher male prevalence of the adult metabolic syndrome? This kind of clear difference in the response to pediatric obesity treatment between sexes has not been described thoroughly in previous reports and warrants further research.

Our results emphasize early treatment and prevention of childhood obesity, a need for new treatment strategies, especially for boys and for those who already have several clustered CV risk factors related to childhood obesity.

## Author contributions

MDV, TL, and JJ: designed the study; MDV, HP, PN, and JJ: performed collection of the data; MDV: handled and analyzed the data, wrote the first and the final draft; MDV, TL, and JJ revised the manuscript. All the authors discussed the data and accepted the final draft of the manuscript.

### Conflict of interest statement

The authors declare that the research was conducted in the absence of any commercial or financial relationships that could be construed as a potential conflict of interest.
